# MDMX is essential for the regulation of p53 protein levels in the absence of a functional MDM2 C-terminal tail

**DOI:** 10.1186/s12860-021-00385-3

**Published:** 2021-09-22

**Authors:** Jack D. Sanford, Jing Yang, Jing Han, Laura A. Tollini, Aiwen Jin, Yanping Zhang

**Affiliations:** 1 Department of Radiation Oncology, University of North Carolina at Chapel Hill, Chapel Hill, NC 27514 USA; 2 Lineberger Comprehensive Cancer Center, University of North Carolina at Chapel Hill, Chapel Hill, NC 27514 USA; 3 Curriculum in Genetics and Molecular Biology, University of North Carolina at Chapel Hill, Chapel Hill, NC 27514 USA; 4 Jiangsu Province Key Laboratory of Immunity and Metabolism and Department of Pathogenic Biology and Immunology, Xuzhou Medical University, Xuzhou, 221002 Jiangsu China; 5 Department of Pharmacology, School of Medicine, University of North Carolina at Chapel Hill, Chapel Hill, NC 27514 USA

**Keywords:** P53, MDM2, MDMX, Cancer, Protein degradation

## Abstract

**Background:**

MDM2 is an E3 ubiquitin ligase that is able to ubiquitinate p53, targeting it for proteasomal degradation. Its homologue MDMX does not have innate E3 activity, but is able to dimerize with MDM2. Although mouse models have demonstrated both MDM2 and MDMX are individually essential for p53 regulation, the significance of MDM2-MDMX heterodimerization is only partially understood and sometimes controversial. MDM2^C462A^ mice, where the C462A mutation abolishes MDM2 E3 ligase activity as well as its ability to dimerize with MDMX, die during embryogenesis. In contrast, the MDM2^Y487A^ mice, where the Y487A mutation at MDM2 C-terminus significantly reduces its E3 ligase activity without disrupting MDM2-MDMX binding, survive normally even though p53 is expressed to high levels. This indicates that the MDM2-MDMX heterodimerization plays a critical role in the regulation of p53. However, it remains unclear whether MDMX is essential for the regulation of p53 protein levels in the context of an endogenous MDM2 C-terminal tail mutation.

**Results:**

Here, we studied the significance of MDM2-MDMX binding in an MDM2 E3 ligase deficient context using the MDM2^Y487A^ mouse embryonic fibroblast (MEF) cells. Surprisingly, down-regulation of MDMX in MDM2^Y487A^ MEFs resulted in a significant increase of p53 protein levels. Conversely, ectopic overexpression of MDMX reduced p53 protein levels in MDM2^Y487A^ MEFs. Mutations of the RING domain of MDMX prevented MDMX-MDM2 binding, and ablated MDMX-mediated suppression of p53 protein expression. Additionally, DNA damage treatment and nuclear sequestration of MDMX inhibited MDMX activity to suppress p53 protein expression.

**Conclusions:**

These results suggest that MDMX plays a key role in suppressing p53 protein expression in the absence of normal MDM2 E3 ligase activity. We found that the ability of MDMX to suppress p53 levels requires MDM2 binding and its cytoplasmic localization, and this ability is abrogated by DNA damage. Hence, MDMX is essential for the regulation of p53 protein levels in the context of an MDM2 C-terminal mutation that disrupts its E3 ligase activity but not MDMX binding. Our study is the first to examine the role of MDMX in the regulation of p53 in the context of endogenous MDM2 C-terminal mutant MEF cells.

**Supplementary Information:**

The online version contains supplementary material available at 10.1186/s12860-021-00385-3.

## Background

p53 is a critical tumor suppressor that is frequently mutated in human cancers [1]. p53 can suppress tumorigenesis through multiple mechanisms, including induction of cell cycle arrest and apoptosis, regulation of the DNA damage response, and regulation of cellular metabolism [1]. p53 function can also be lost through overexpression or genetic amplification of endogenous suppressors of p53, chiefly MDM2 and MDMX. Deletion of either MDM2 or MDMX in mice results in unregulated p53 activity that leads to early embryonic lethality [2–4]. Although it is clear that MDM2 and MDMX antagonize p53 function, the mechanism of action of these proteins remains only partially understood.

MDM2 is a RING-type E3 ubiquitin ligase that is able to ubiquitinate p53, targeting it for proteosomal degradation [5–7]. MDM2 is also able to inhibit p53 transcriptional activity by binding to the N-terminal transcriptional activating domain (TAD) of p53 [8]. Although MDMX also has a RING domain, biochemical studies have shown that MDMX lacks innate E3 ligase activity [9]. Similar to MDM2, MDMX has been shown to bind p53 and inhibit its transcriptional activity [10]. MDM2 and MDMX can interact with each other through their respective RING domains [11]. Recent structural studies have shown that MDM2 dimerization is required for its E3 ligase activity [12, 13]. MDM2 is able to form both MDM2-MDM2 homodimers, as well as MDM2-MDMX heterodimers [13–16]. However, the consequences MDM2-MDMX heterodimerization remain unclear. One major point of confusion lies in conflicting in vitro data. For example, studies have shown that MDMX is able to either inhibit MDM2 E3 ligase activity [9] or promote MDM2 E3 ligase activity [17].

In order to elucidate the role of MDM2-MDMX dimerization in the regulation of p53 under physiological conditions, we generated MDM2^C462A^ and MDM2^Y487A^ mutant mice. The MDM2^C462A^ mutation disrupts MDM2 RING function, preventing MDM2-MDMX heterodimer formation and inhibiting MDM2 E3 ligase activity. The MDM2^C462A^ mouse embryos are not viable, indicating that proper structure of the MDM2 RING domain is essential for the regulation of p53 during early embryonic development [18]. The MDM2^Y487A^ mutation, on the other hand, ablates MDM2 E3 ligase activity without interrupting MDM2-MDMX heterodimer formation [19–21], allowing for separate analysis of the relative importance of heterodimerization and E3 ligase activity. Surprisingly, the MDM2^Y487A^ mice were viable and developmentally normal despite expressing high levels of p53, but the mice were hypersensitive to DNA damage. These results suggest that the MDM2-MDMX heterodimerization is critical for suppressing p53 activity for normal development, while MDM2 E3 ligase activity is indispensable for recovery from DNA damage [21]. However, it remains unclear whether MDMX is essential for the regulation of p53 protein levels in the context of an endogenous MDM2 C-terminal tail mutation that disrupts MDM2 E3 ligase function but not MDM2-MDMX heterodimerization. In this paper, we address this knowledge gap by examining the role of MDMX in the regulation of p53 in the context of endogenous MDM2^Y487A^. We find that MDMX is essential for the regulation of p53 protein levels in MDM2^Y487A^ MEFs, even in the absence of detectable MDM2 E3 ligase activity. Furthermore, we find that this activity is localized to the cytoplasm and is ablated by DNA damage.

## Results

### Down-regulation of MDMX in MDM2^487^ MEFs increases p53 protein levels

**Fig. 1:**
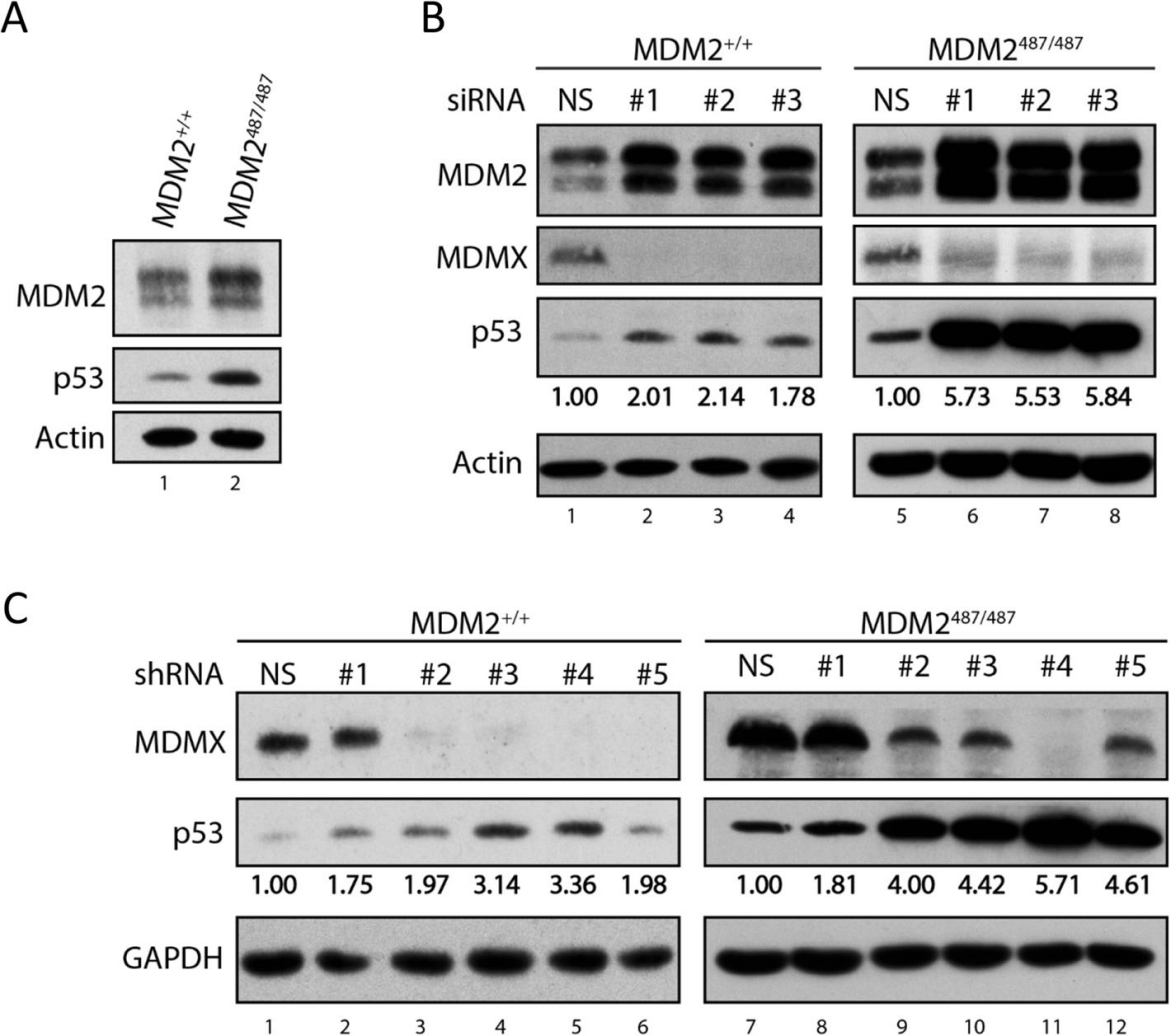
MDMX suppresses p53 protein levels in MDM2487/487 MEFs. **A** WT (MDM2+/+) and MDM2487/487 MEFs were lysed and subject to immunoblotting for the indicated proteins. **B** WT and MDM2487/487 MEFs were treated with nonspecific (NS) siRNA, or MDMX-targeting siRNA for 32 h prior to lysis and immunoblotting. **C** WT and MDM2487/487 MEFs were transduced with nonspecific (NS), or MDMX-targeting shRNA lentiviral constructs for 48 h in the presence of 1μg/mL puromycin prior to lysis and immunoblotting

As previously shown, the MDM2^Y487A^ mutation greatly inhibits the E3 ligase activity of MDM2 [19–21]. Mouse embryonic fibroblast (MEF) cells generated from MDM2^Y487A/Y487A^ mice (MDM2^487/487^ hereafter) display higher p53 protein levels than WT MDM2 MEFs (Fig. 1A). This occurs even though MDM2^487/487^ MEFs have higher levels of endogenous MDM2 compared to WT MEFs. We wondered whether MDMX plays any role in the regulation of p53 protein levels in the MDM2^487/487^ MEFs. In order to test this, we knocked down MDMX using siRNA in WT and MDM2^487/487^ MEFs and examined p53 protein levels. Knockdown of MDMX resulted in an increase in p53 protein levels in WT MEFs, indicating MDMX negatively regulates p53 protein levels in the context of WT MDM2 (Fig. 1B, left). Surprisingly, knockdown of MDMX in MDM2^487/487^ MEFs resulted in an even greater increase in p53 protein levels than was observed in the WT MEFs (Fig. 1B, right). While knockdown of MDMX in WT MEFs resulted in about a two-fold increase in p53 levels, knockdown of MDMX in MDM2^487/487^ MEFs resulted in more than a five-fold increase in p53 levels (Fig. 1B). To validate the siRNA knockdown results, we utilized lentiviral-guided shRNA targeting MDMX. Consistent with the siRNA results, shRNA-mediated MDMX knockdown resulted in moderate increase in p53 levels in WT MEFs and more pronounced increases in p53 levels in MDM2^487/487^ MEFs (Fig. 1C). Importantly, the degree of p53 protein increase in MDM2^487/487^ MEFs inversely correlated with the degree of MDMX knockdown, indicating a causal relationship. These results show that despite the increased stability of p53 in the MDM2^487/487^ MEFs, p53 protein levels can be further increased by MDMX knockdown, suggesting that MDMX suppresses p53 protein expression in the absence of detectable MDM2 E3 activity.

### Overexpression of MDMX in MDM2^487/487^ MEFs decreases p53 protein levels

**Fig. 2:**
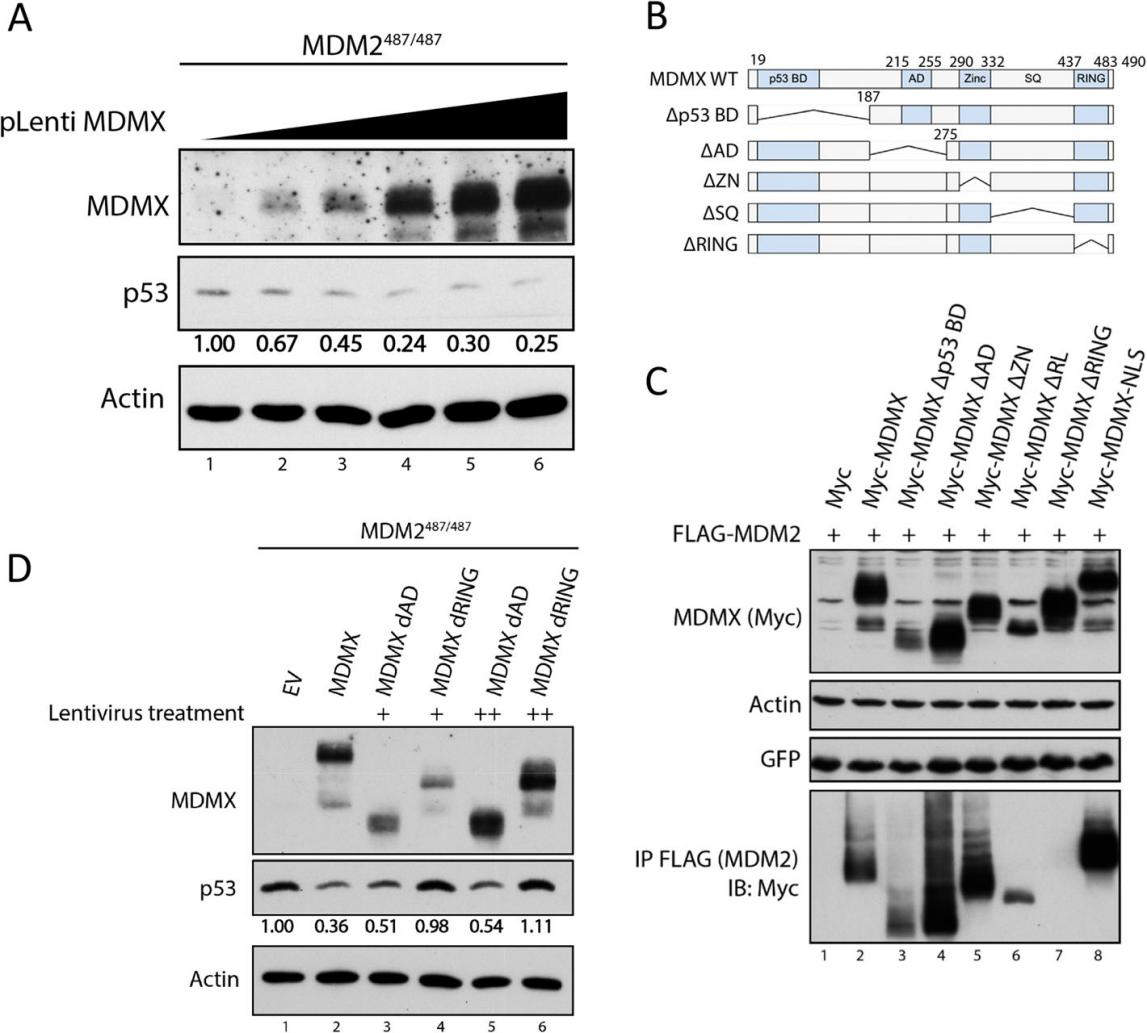
MDM2-MDMX binding is essential for MDMX-mediated suppression of p53 protein levels. **A** MDM2487/487 MEF cells were treated with increasing volumes of MDMX-expressing lentiviral constructs for 48 h prior to lysis and immunoblotting. **B**, **C** HEK293 cells were transfected with GFP vector and FLAG-MDM2 along with Myc-tagged MDMX constructs for 24 h prior to lysis and immunoprecipitation (IP). 1 mg of lysate was subject to IP using anti-FLAG beads prior to immunoblotting using an anti-Myc antibody. **D** MDM2487/487 MEFs were treated with empty vector (EV), lentiviral constructs expressing full-length MDMX, acidic binding domain deletion (ΔAD), or RING domain deletion (ΔRING) MDMX for 48 h prior to lysis and immunoblotting

To further validate the above observation of MDMX suppressing p53 protein expression, we utilized a lentiviral system to overexpress MDMX in the MEF cell system. In order to more accurately investigate the relationship between MDMX protein levels and p53 protein levels, we first performed a titration experiment in which increasing doses of MDMX lentivirus were added to MDM2^487/487^ MEF cells. Increasing MDMX overexpression resulted in a dose-dependent decrease in p53 protein levels, suggesting a causal relationship (Fig. 2A). Continued increase of MDMX overexpression did not result in complete eliminating p53 protein expression, suggesting that MDMX is not the only rate-limiting factor for the regulation of p53 protein levels in this system (Fig. 2A, lanes 4–6). Previous studies have shown that MDMX overexpression is able to reactivate the E3 ligase activity of MDM2^Y489A^ mutant [19, 20]. Therefore, we hypothesized that the endogenous MDM2^Y487A^ is a rate-limiting factor and MDMX overexpression reactivates MDM2^Y487A^ E3 ligase activity. To test this hypothesis, we investigated whether MDMX-mediated suppression of p53 expression was dependent on its ability to bind MDM2. We first generated a number of MDMX deletion mutants (Fig. 2B), and assessed their MDM2-binding activity. Consistent with previous studies showing MDM2 and MDMX interact through their respective RING domains [11–13], deletion of MDMX RING domain (MDMX^ΔRING^), but not other regions of MDMX, prevented MDM2-MDMX binding (Fig. 2C). We then tested the ability of these MDMX deletion mutants to suppress p53 using MDMX^ΔAD^, which maintains MDM2 binding, and MDMX^ΔRING^, which does not bind MDM2. Overexpression of full-length MDMX and MDMX^ΔAD^ was able to suppress p53 protein levels to a similar degree, while overexpression of MDMX^ΔRING^ was unable to inhibit p53 protein expression (Fig. 2D), indicating that MDM2-MDMX binding is necessary for MDMX to suppress p53 expression in MDM2^487/487^ MEFs. Together, these data show that down-regulation of MDMX increases p53 levels while overexpression of MDMX decreases p53 levels in the E3 defective MDM2^487/487^ MEFs, and the ability of MDMX to regulate p53 levels depends on MDM2 binding.

### DNA damage inhibits MDMX suppression of p53 protein expression

**Fig. 3:**
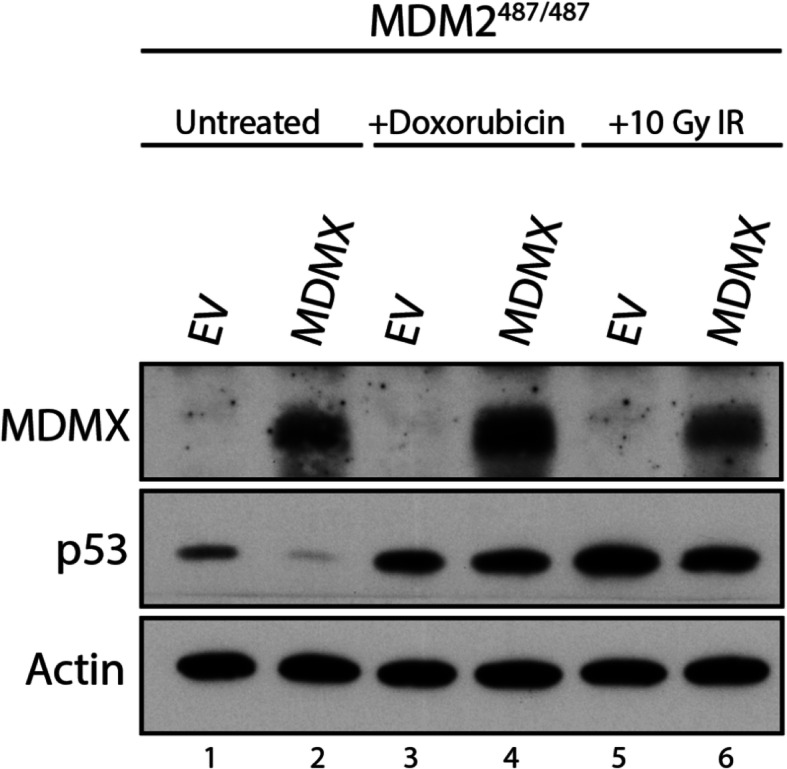
DNA damage inhibits MDMX suppression of p53 protein expression. MDM2487/487 MEFs were treated with empty vector (EV) or MDMX-expression lentiviral constructs for 48 h prior to lysis. For DNA damage conditions, MEF cells were treated with 1μg/mL doxorubicin for four hours, or 10 Gy ionizing irradiation (IR) for six hours prior to lysis and immunoblotting

Studies have shown that DNA damage increases p53 protein stability and activity through a variety of mechanisms. DNA damage promotes p53 phosphorylation that decreases the affinity of MDM2 and MDMX for p53 [22]. DNA damage also promotes phosphorylation of MDM2 and MDMX in a manner that decreases their ability to inhibit p53 [23–25]. In order to determine whether DNA damage affects the ability of MDMX to suppress p53 expression in the MDM2^487/487^ context, MDM2^487/487^ MEF cells were infected with empty lentivirus vector (EV) or MDMX lentivirus. The MEFs were then left untreated, or treated with 1μg/mL doxorubicin or 10 Gy ionizing irradiation to cause DNA damage. While MDMX strongly inhibited p53 protein expression in untreated MDM2^487/487^ MEFs, doxorubicin and ionizing radiation treatment ablated MDMX activity, suggesting that MDMX activity to suppress p53 levels is inhibited by DNA damage treatment in this context (Fig. 3).

### The p53-suppressing activity of MDMX is localized to the cytoplasm

**Fig. 4:**
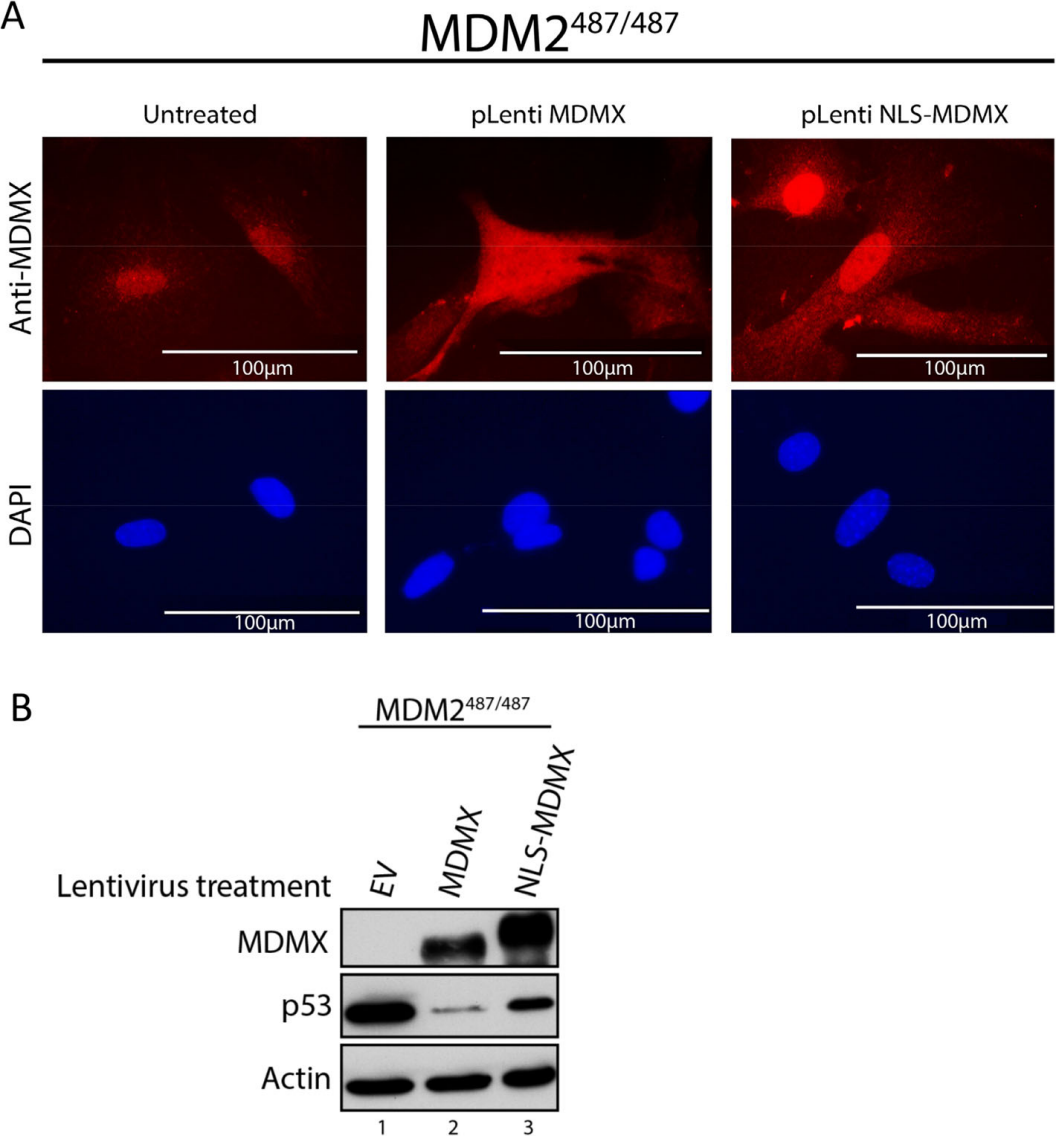
Nuclear sequestration reduces MDMX activity to suppress p53 protein expression. **A** MDM2487/487 MEF cells were treated with empty vector (EV), MDMX or NLS-tagged MDMX lentiviral constructs for 48 h prior to immunostaining using an anti-MDMX antibody, and DAPI nuclear staining. **B** MDM2487/487 MEFs were treated with empty vector (EV), MDMX, or NLS-tagged MDMX lentiviral constructs for 48 h prior to lysis and immunoblotting

DNA damage results in translocation of MDMX from the cytoplasm to the nucleus [26–28]. It has been hypothesized that nuclear translocation of MDMX results in its degradation by MDM2 [24, 27]. Alternatively, it has been proposed that nuclear translocation of MDMX inhibits MDM2 E3 ligase activity, leading to p53 stabilization [9, 29]. In order to determine the effect of nuclear localization of MDMX on its ability to suppress p53 protein levels, we attached an SV40 nuclear localization signal (NLS) sequence to the N-terminus of MDMX to generate the NLS-MDMX lentivirus. We overexpressed NLS-MDMX in MDM2^487/487^ MEFs and examined their subcellular localization. Immunofluorescent staining indicated that WT MDMX localizes predominantly to the cytoplasm, while NLS-MDMX localizes predominantly to the nucleus (Fig. 4A). Interestingly, NLS-MDMX overexpression resulted in a much weaker effect on suppressing p53 protein levels, despite a higher level of NLS-MDMX expression than that of WT MDMX in this experiment (Fig. 4B), suggesting that the activity of MDMX to suppress p53 protein expression is localized to the cytoplasm. Together, our results suggest that MDMX suppresses p53 protein levels in MDM2^487/487^ MEFs in an MDM2 binding- and cytoplasmic-localization dependent manner.

## Discussion

Recent biochemical studies have shown that the C-terminal tail, downstream of the RING domain, is essential for MDM2 E3 ligase activity, and that mutation of C-terminal amino acids of MDM2, including Y487 (Y489 in human MDM2), ablates MDM2 E3 ligase activity, and that MDMX overexpression can restore the E3 ligase activity of C-terminal mutant MDM2 [13, 16, 19, 20]. These studies suggest that successful polyubiquitination of p53 requires an intact MDM2 C-terminal tail. Our data are consistent with this model. We have shown that in MDM2^487/487^ MEFs, MDMX knockdown results in a great increase in p53 protein levels (Fig. 1), despite the fact that p53 protein levels in MDM2^487/487^ MEFs were already significantly higher than in their WT MEF counterparts. This suggests that MDM2^487/487^ MEFs are more dependent on MDMX for the regulation of p53 protein levels than WT MEFs. One explanation for this observation is that in WT MEFs, MDM2 homodimers are able to regulate p53 protein levels in the absence of MDMX. In MDM2^487/487^ MEFs, however, there is no available functional MDM2 C-terminal tail, and therefore MDM2^487/487^ homodimers cannot polyubiquitinate p53, increasing reliance on MDMX for the degradation of p53. This model is consistent with a previous study where the RING domain of MDMX was deleted [30]. In the study, p53 degradation was shown to occur at a similar rate in MDMX^ΔRING^ MEFs as in MDMX^+/+^ MEFs, indicating that in a MEF cell system MDM2 can degrade p53 in the absence of MDM2-MDMX heterodimerization [30]. However, this study uses exogenous p53, as MDMX^ΔRING^ MEFs are nonviable with endogenous p53.

In the current study, we showed that MDMX overexpression results in a decrease in p53 protein levels in the context of E3 ligase deficient MDM2^487/487^ MEFs. However, in an MDMX overexpression titration experiment, the maximal effect of MDMX overexpression was reached using a relatively low dose of MDMX lentivirus (Fig. 2A). This suggests that endogenous factors, other than ectopically overexpressed MDMX, play a rate-limiting role for suppression of p53 protein levels. Deletion of the RING domain of MDMX ablates the ability of MDMX to suppress p53 protein levels, suggesting that such a function of MDMX relies on interaction with MDM2 (Fig. 2C). One possible explanation for these data is that MDMX is able to restore MDM2^Y487A^ E3 ligase activity by increasing the ratio of functional MDM2-MDMX heterodimers relative to nonfunctional MDM2^Y487A^ homodimers. Previous studies have shown that supplementation of MDMX to MDM2 C-terminal tail mutations results in reactivation of MDM2 E3 ligase activity [13, 16, 19, 20]. Our study supports this notion in a context of endogenous MDM2^Y487A^.

The role of MDMX subcellular localization in p53 regulation remains poorly understood and controversial. While p53 and MDM2 are both primarily localized to the nucleus, MDMX is primarily localized to the cytoplasm. Previous studies have shown that DNA damage leads to nuclear import of MDMX [26, 27]. Several hypotheses have emerged to explain the nuclear translocation of MDMX. One such hypothesis is that MDMX nuclear import leads to the degradation of MDMX by MDM2 [27]. It has also been suggested that MDMX can increase p53 stability by inhibiting MDM2 following DNA damage and nuclear translocation [9, 29]. Our data show that nuclear sequestration inhibits the capacity of MDMX to suppress p53 in MDM2^487/487^ MEFs (Fig. 4). This is consistent with previous studies showing that p53 polyubiquitination and degradation primarily occurs in the cytoplasm, and that blocking p53 nuclear export using leptomycin B (LMB) results in decreased p53 polyubiquitination and degradation [31]. Previous studies have shown that nuclear export of p53 is essential for p53 degradation, suggesting that p53 is not normally degraded by nuclear proteasomes [32, 33]. Therefore, if p53 polyubiquitination occurs in the nucleus, as it would in the context of NLS-MDMX nuclear sequestration, polyubiquitinated p53 would have to travel to the cytoplasm prior to being degraded by cytoplasmic proteasomes. This would leave p53 more susceptible to cytoplasmic deubiquitinases, such as USP10, than it would be if both polyubiquitination and proteasomal degradation occurred simultaneously in the cytoplasm [34]. Thus, nuclear import of MDMX could increase p53 stability by increasing the distance between the site of polyubiquitination and the site of degradation. It is also possible that MDM2-MDMX heterodimers have reduced activity in the nucleus, or that nuclear sequestration of MDMX also results in nuclear sequestration of polyubiquitinated p53, inhibiting its degradation. Future studies are required to determine the mechanism through which nuclear sequestration of MDMX inhibits its function.

## Conclusions

This study shows that MDMX is essential for the regulation of p53 protein levels in the context of an endogenous C-terminal tail mutant MDM2. Furthermore, MDMX activity in this context relies on MDM2-MDMX binding, is inhibited by DNA damage, and is localized to the cytoplasm. These results have implications for the function of MDM2-MDMX heterodimers and the viability of the MDM2^Y487A/Y487A^ mutant mouse model.

### Experimental procedures

#### Cell culture and reagents

Mouse embryonic fibroblasts (MEF) were generated from MDM2^Y487A/Y487A^ and MDM2^+/+^ embryos and passage 0 MEF cells were frozen down in liquid nitrogen for future use. MEF cells were cultured in a humidified 37 °C, 5% CO_2_, 3% O_2_ incubator. Cells were cultured in Dulbecco’s modified Eagle’s medium (Sigma) supplied with 10% fetal bovine serum (Sigma) and penicillin (100 IU/ml)/streptomycin (Sigma, 100 mg/ml). HEK293 cells were obtained through the ATCC and cultured in a humidified 37 °C, 5% CO_2_ incubator in Dulbecco’s modified Eagle’s medium (Sigma) supplied with 10% fetal bovine serum (Sigma) and penicillin (100 IU/ml)/streptomycin (Sigma, 100 mg/ml).

#### Antibodies

The following primary antibodies were used for western blot analysis: mouse anti-actin (MAB1501, Chemicon), mouse anti-p53 (pAb122), mouse anti-MDM2 (2A10), mouse anti-MDMX (Sigma MDMX Clone-82, M0445), mouse anti-GAPDH (ThermoFisher, MA5–15738), rabbit anti-Myc (gift from Yue Xiong), mouse anti-GFP (NeoMarkers, clone AB-2). The following antibody was used for immunofluorescent staining: mouse anti-MDMX (Clone 7A8, gift from Jiandong Chen).

#### siRNA treatment

Cells were treated with 20 μM of siRNA using the Lipofectamine RNAiMax reagent (ThermoFisher, catalog #13778150). Cells were analyzed 32–36 h after siRNA transfection. MDMX-targeting siRNA duplexes were purchased from Invitrogen (Stealth RNAi siRNA Duplex, siRNA 1 ID: MSS206594, siRNA 2 ID: MSS206595, siRNA 3 ID: MSS206596).

#### Western blotting

For western blot analysis, cells were lysed in 0.5% Nonidet P-40 buffer containing protease inhibitor mixture (leupeptin, catalog no. L2884; aprotinin, catalog no. A1155; benzamidine, catalog no. B6506; and trypsin inhibitor, catalog no. T9003; all from Sigma), 1 mM PMSF (Sigma, catalog no. P7626), 1 mM NaVO3 (Fisher Scientific, catalog no. S454–50), and 1 mM DTT (Roche, catalog no. 03117014001) for 30 min. 100-150 μg of protein was separated on a 12.5% SDS-PAGE gel prior to transfer to a nitrocellulose membrane (BioRad). Transfers were assessed by staining the membranes with Ponceau S (Sigma, catalog no. P3504) for 5 min, followed by several brief washes with double-distilled H_2_O. Membranes were blocked for at least 1 h in phosphate-buffered saline containing 5% nonfat milk and 0.1% Tween 20. Membranes were then incubated with the appropriate primary antibody diluted in blocking buffer for 2 h to overnight. Membranes were washed three times in phosphate-buffered saline containing 0.1% Tween 20 and then incubated with the appropriate HRP-conjugated secondary antibody diluted in blocking buffer for 1 h. Membranes were washed four times in PBS-T and then developed with Supersignal West Pico chemiluminescent substrate or Supersignal West Dura chemiluminescent substrate according to the instructions of the manufacturer (Pierce, catalog no. 34080 and 34,075).

#### Lentiviral transduction

Lentiviral overexpression constructs were generated using the gateway cloning system to insert MDMX constructs into the pLenti4 puro-DEST vector. Vector sequences were verified by DNA sequencing both before and after gateway insertion. For shRNA treatments, MDMX-targeting or nonspecific shRNAs were inserted into the pLKO.1 vector and verified by DNA sequencing. The following shRNA sequences were used to target MDMX: MDMX shRNA 1: GCAAGAAGTTTAATTCTCCAA, MDMX shRNA 2: GCAGAATTTCTTCGGAACAAA, MDMX shRNA 3: CCCGATTGTAGGAGAACCATT, MDMX shRNA 4: CTCAACTGATTTACAGACAAA, MDMX shRNA 5: GCGCGAGAGAACAAACAGATA. Lentiviral particles were generated by the UNC Lenti-shRNA Core Facility. For lentiviral transduction, cells were plated at 60% confluence in a 6-well plate and treated with 25-200 μL of lentiviral supernatant in 1 mL total media containing 8 μg/mL hexadimethrine bromide (polybrene, Sigma catalog #107689). Cells were incubated with lentiviral particles for 48 h prior to lysis for protein analysis.

#### Immunoprecipitation

HEK293 cells were plated at 70% confluence in a 60 mm dish and then transfected with 1 μg of plasmid mixture using the Effectene transfection reagent (Qiagen, catalog #301425). 24 h after transfection, cells were lysed in 0.1% nonidet P-40 buffer containing protease inhibitor, PMSF, NaVO3 and DTT. 100 μg of lysate was used as loading control, while 1 mg of lysate was used for immunoprecipitation (IP). Prior to IP, lysates were pre-cleared using 75-100 μL of CL4B beads. IP was performed using anti-Flag M2 affinity gel (Sigma, catalog #A2220) by rotating lysates with 5 μL Flag beads at 4 °C overnight. Beads were then washed three times with 0.1% nonidet P-40 buffer. Precipitated proteins were then isolated in 1X SDS loading buffer and analyzed via western blot.

#### Immunofluorescent staining

MEF cells were plated at low confluence and treated with a low volume (50 μL) of lentiviral particles for 48 h prior to fixation using 10% formalin solution (Sigma, catalog #HT501128). Cells were permeabilized using 0.2% Triton X-100 diluted in phosphate buffered saline for five minutes at 4 °C. Permeabilized cells were then incubated in 0.5% bovine serum albumin (BSA) solution for at least 30 min before overnight exposure to primary antibody solution. Cells were then treated with AlexaFluor 594-conjugated goat anti-mouse secondary antibody (Jackson ImmunoResearch Laboratories) for 30–45 min. Followed by incubating with 4,6-diamidino-2-phenylindole (DAPI) for 2 min for nuclear counterstaining. After staining, cells were analyzed using an Olympus IX-81 microscope fitted with a SPOT camera and software. Signal brightness and contrast was increased equally for all images using ImageJ software for clear signal visualization.

## Supplementary Information

**Additional file 1.**

## Data Availability

All data generated or analysed during this study are included in this published article. Contact the corresponding author regarding availability of reagents and materials used in this study.

## Abbreviations

MDM2: mouse double minute 2
MDMX: mouse double minute 4
MEF: mouse embryonic fibroblast cells
RING: really interesting new gene
NLS: nuclear localization signal
TAD: transcriptional activation domain
HEK293: human embryonic kidney 293 cells
