# Supplementary material for: MDMX is essential for the regulation of p53 protein levels in the absence of a functional MDM2 C-terminal tail
Source: BMC Mol Cell Biol. 2021 Sep 22;22:46. doi: 10.1186/s12860-021-00385-3 (PMC8459461; doi:10.1186/s12860-021-00385-3)
Supplement: Supplementary file 1 — Additional file 1. [file 12860_2021_385_MOESM1_ESM.docx]

**Supplementary image file:**

**MDMX is essential for the regulation of p53 protein levels in the absence of a functional MDM2**

**C-terminal tail**

Jack D. Sanford^1,2,3^, Jing Yang^1,2,5^, Jing Han^1,2,5^, Laura A. Tollini^1,2,3^, Aiwen Jin^1,2^, Yanping Zhang^1,2,3,4,*^

^1^Department of Radiation Oncology, ^2^Lineberger Comprehensive Cancer Center, ^3^Curriculum in Genetics and Molecular Biology, ^4^Department of Pharmacology, School of Medicine, University of North Carolina at Chapel Hill, Chapel Hill, NC 27514, USA.

^5^Jiangsu Province Key Laboratory of Immunity and Metabolism and Department of Pathogenic Biology and Immunology, Xuzhou Medical University, Xuzhou, Jiangsu 221002, China

^*^To whom correspondence should be addressed: ypzhang@med.unc.edu

Supplementary file 1: Original western blot film scans

**Full-length film scans are attached. Most films contain multiple experiments, and thus have been cropped for clarity. Full-length films can be viewed by adjusting the crop dimensions.**

Figure 1a:









Scans of 3 different exposures.

Left lane: WT MEF. Right lane: 487 MEF

Top bands: MDM2

Upper middle bands: MDMX

Lower middle bands: p53

Lowest bands: Actin

**Figure 1: MDMX suppresses p53 protein levels in MDM2^487/487^ MEFs.** (A) WT (MDM2^+/+^) and MDM2^487/487^ MEFs were lysed and subject to immunoblotting for the indicated proteins. (B) WT and MDM2^487/487^ MEFs were treated with nonspecific (NS) siRNA, or MDMX-targeting siRNA for 32 hours prior to lysis and immunoblotting. (C) WT and MDM2^487/487^ MEFs were transduced with nonspecific (NS), or MDMX-targeting shRNA lentiviral constructs for 48 hours in the presence of 1ug/mL puromycin prior to lysis and immunoblotting.

Figure 1b:

Wild-type MEF cells:





Lanes (left to right): siNS, siMDMX 1, siMDMX 2, siMDMX 3

Actin bands

Lanes (left to right): siNS, siMDMX 1, siMDMX 2, siMDMX 3

Top bands: MDM2

Middle bands: MDMX

Lowest bands: p53





MDM2 487 MEF cells.







Scans of 2 different exposures:

Lanes (left to right): siNS, siMDMX 1, siMDMX 2, siMDMX 3

Top bands: MDM2

Upper middle bands: MDMX

Lower middle bands: p53

Lowest bands: Actin

Figure 1c:

Wild-type MEF cells





Scans of 2 different exposures:

Lanes (left to right): MDMX -/- MEF cells, shNS, shMDMX 1, shMDMX 2, shMDMX 3, shMDMX 4, shMDMX 5

Top bands: MDMX

Middle bands: p53

Lowest bands: Actin








Lanes (left to right): MDMX -/- MEF cells, shNS, shMDMX 1, shMDMX 2, shMDMX 3, shMDMX 4, shMDMX 5

Bands: GAPDH

MDM2 487 MEF cells





Scans of 2 different exposures:

Lanes (left to right): shNS, shMDMX 1, shMDMX 2, shMDMX 3, shMDMX 4, shMDMX 5

Top Bands: MDMX

Middle bands: p53

Lowest bands: GAPDH





Figure 2a:








Scans of 2 different exposures:

Lanes (left to right): pLenti MDMX 10uL, pLenti MDMX 25uL, pLenti MDMX 50uL, pLenti MDMX 100 uL, pLenti MDMX 150 uL, pLenti MDMX 200uL

Top bands: MDMX

Middle bands: p53

Lowest bands: Actin

**Figure 2: MDM2-MDMX binding is essential for MDMX-mediated suppression of p53 protein levels.** (A) MDM2^487/487^ MEF cells were treated with increasing volumes of MDMX-expressing lentiviral constructs for 48 hours prior to lysis and immunoblotting. (B-C) HEK293 cells were transfected with GFP vector and FLAG-MDM2 along with Myc-tagged MDMX constructs for 24 hours prior to lysis and immunoprecipitation (IP). 1mg of lysate was subject to IP using anti-FLAG beads prior to immunoblotting using an anti-Myc antibody. (D) MDM2^487/487^ MEFs were treated with empty vector (EV), lentiviral constructs expressing full-length MDMX, acidic binding domain deletion (ΔAD), or RING domain deletion (ΔRING) MDMX for 48 hours prior to lysis and immunoblotting.

Figure 2c:

Loading


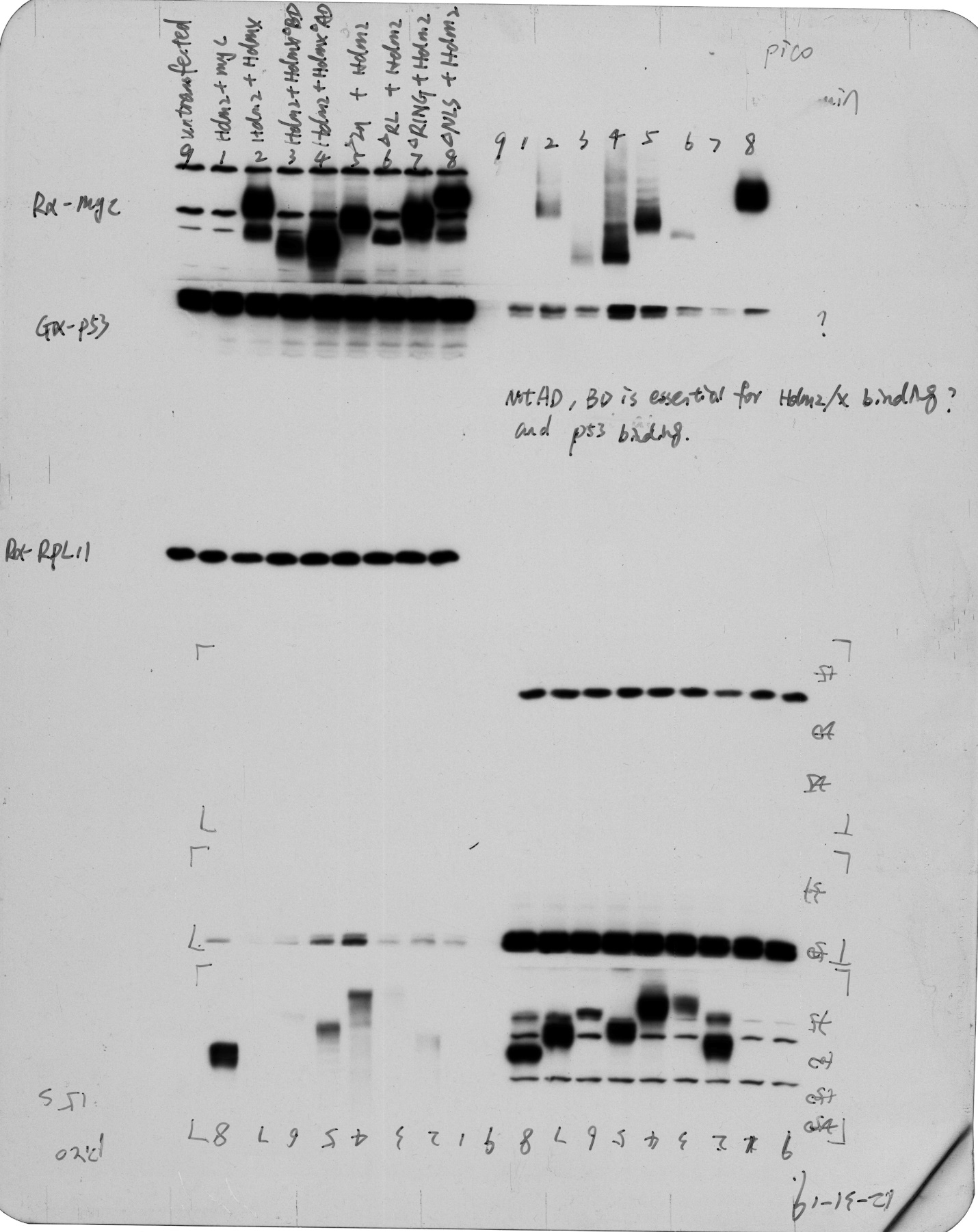


Scans of 3 different films from the same membranes:

Lanes (left to right): untransfected, Myc, Myc-MDMX, Myc-MDMX dBD, Myc-MDMX dAD, Myc-MDMX dZN, Myc-MDMX dRL, Myc-MDMX dRING, Myc-MDMX-NLS

Top film: Myc (MDMX) loading

Middle film:

Top bands: Myc (MDMX) reblot)

Middle bands: Actin

Lowest bands: GFP


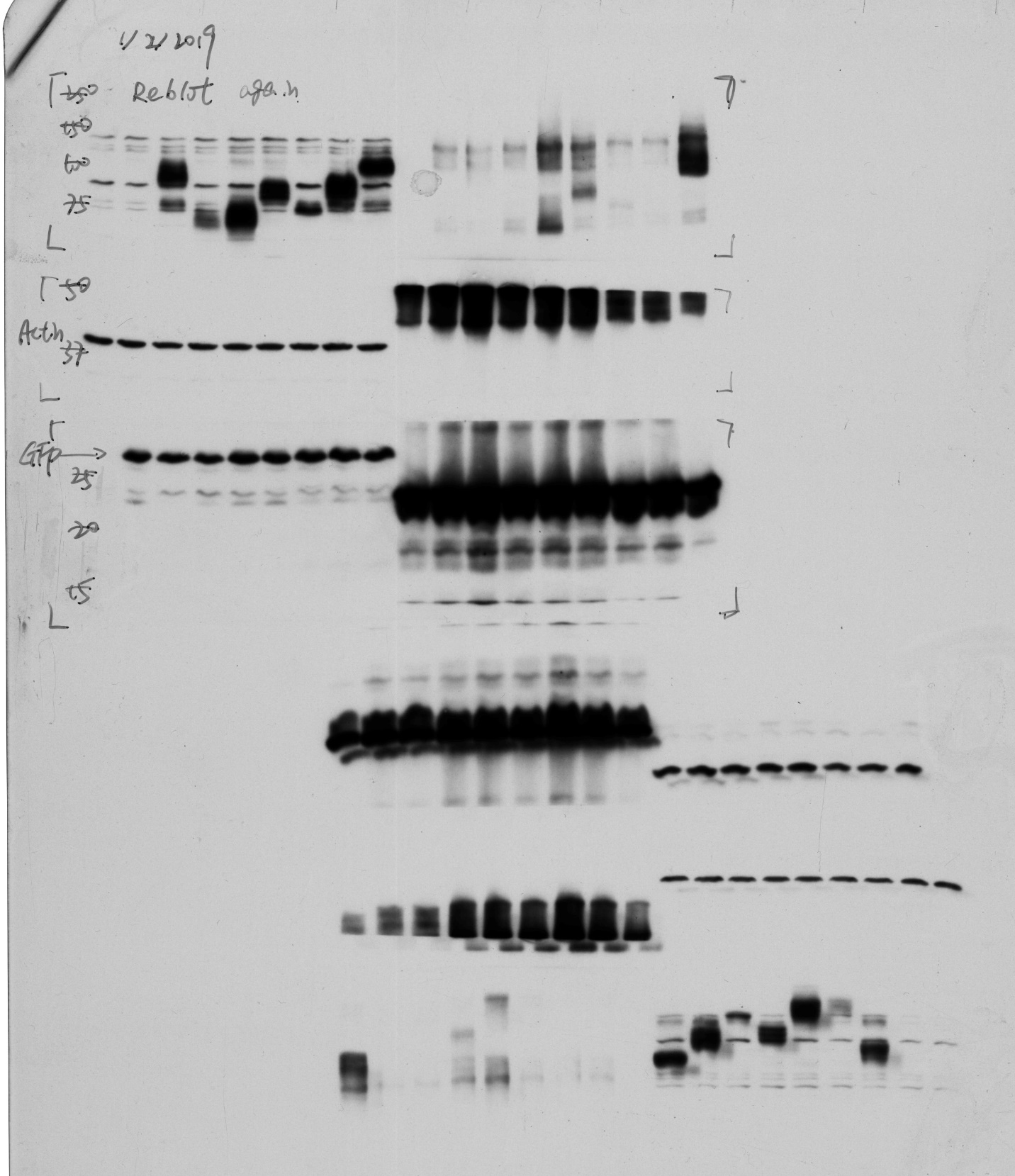


Flag IP


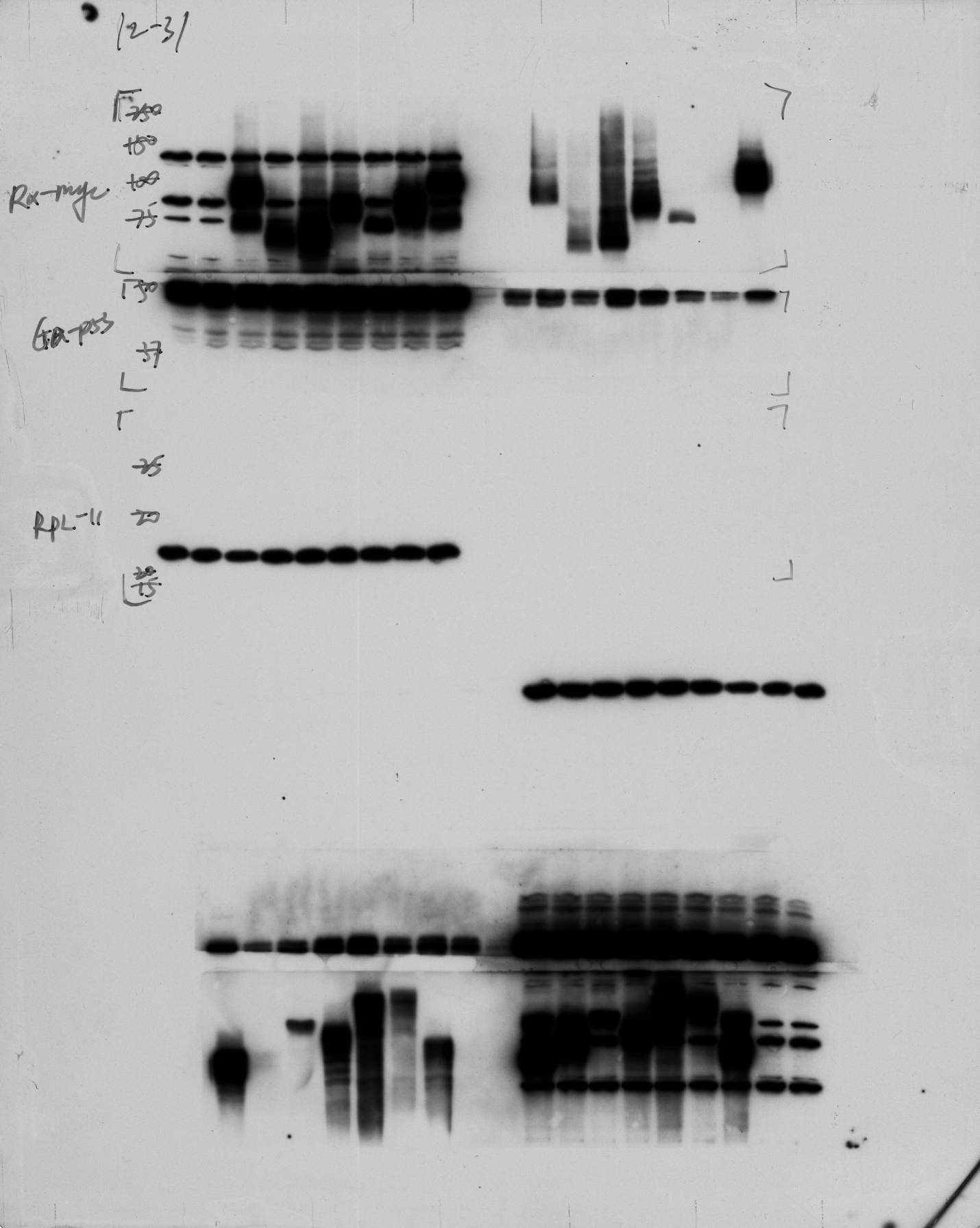


Lanes (left to right): untransfected, Myc, Myc-MDMX, Myc-MDMX dBD, Myc-MDMX dAD, Myc-MDMX dZN, Myc-MDMX dRL, Myc-MDMX dRING, Myc-MDMX-NLS

Flag IP, IB for Myc

Figure 2d:





Scans of 2 different exposures:

Lanes (left to right): pLenti EV, pLenti MDMX, pLenti MDMX dAD 25uL, pLenti MDMX dRING 25uL, pLenti MDMX dAD 50 uL, pLenti MDMX dRING 50uL

Top bands: MDMX

Middle bands: p53

Lowest bands: Actin





Figure 3:





Scans of 2 different exposures:

Lanes (left to right): pLenti EV untreated, pLenti HDMX untreated, pLenti EV + IR, pLenti HDMX + IR, pLenti EV + Doxorubicin, pLenti HDMX + Doxorubicin

Top bands: MDMX

Middle bands: p53

Lowest bands: Actin





**Figure 3: DNA damage inhibits MDMX suppression of p53 protein expression.** MDM2^487/487^ MEFs were treated with empty vector (EV) or MDMX-expression lentiviral constructs for 48 hours prior to lysis. For DNA damage conditions, MEF cells were treated with 1ug/mL doxorubicin for four hours, or 10 Gy ionizing irradiation (IR) for six hours prior to lysis and immunoblotting.

Figure 4b:





Lanes (left to right): pLenti EV, pLenti MDMX, pLenti NLS-MDMX

Top bands: MDMX

Middle bands: p53

Lowest bands: Actin

**Figure 4: Nuclear sequestration reduces MDMX activity to suppress p53 protein expression.** (A) MDM2^487/487^ MEF cells were treated with empty vector (EV), MDMX or NLS-tagged MDMX lentiviral constructs for 48 hours prior to immunostaining using an anti-MDMX antibody, and DAPI nuclear staining. (B) MDM2^487/487^ MEFs were treated with empty vector (EV), MDMX, or NLS-tagged MDMX lentiviral constructs for 48 hours prior to lysis and immunoblotting.
